# Speeding up maximum population transfer in periodically driven multi-level quantum systems

**DOI:** 10.1038/s41598-019-52595-7

**Published:** 2019-11-07

**Authors:** Sebastián Carrasco, José Rogan, Juan Alejandro Valdivia

**Affiliations:** 1 Departamento de Física, Facultad de Ciencias, Universidad de Chile, Casilla 653, Santiago, 7800024 Chile; 2 Centro para la Nanociencia y la Nanotecnolgía, CEDENNA, Santiago, 9170124 Chile

**Keywords:** Quantum mechanics, Quantum information

## Abstract

A fast and robust approach to controlling the quantum state of a multi-level quantum system is investigated using a twofrequency time-varying potential. A comparison with other related approaches in the context of a two-level system is also presented, showing similar times and fidelities. As a concrete example, we study the problem of a particle in a box with a periodically oscillating infinite square-well potential, from which we obtain results that can be applied to systems with periodically oscillating boundary conditions. We show that the transition between the ground and first excited state is about 20 times faster than the one performed using a single frequency, both with fidelity of 99.97%. The transition time is about 3.5 times the minimum allowed by quantum mechanics. A test of the robustness of the approach is presented, concluding that, counter-intuitively, it is not only faster but also easier to tune up two frequencies than one. This robustness makes the approach suitable for real applications.

## Introduction

The study of the time dependence in quantum systems, and in particular its applications for its control in a systematic an efficient manner, is a topic at the forefront of scientific research^1–5^. As an example, Doescher and Rice were the first to propose the problem of the infinite square well potential with moving wells, which was then analyzed with different approximations in several publications^1–4^. Recently, researchers have shown, using an analytical exacta approach, that it is possible to control the transition between two predefined states by a particular time-dependent wall motion of this system^6^, which extended the results obtained by Lenz *et al*.^7^ for a different system. It is possible to derive analytical expressions that characterize this quantum variant of the classical Fermi acceleration problem^8^, and that has applicability in other systems that follow equations that are similar to Schroedinger’s, e.g., Bose-Einstein condensates, quantum, and classical optical systems, fluids, etc.

Hence, the problem of controlling the system has been systematically addressed, now we will address the efficiency of the process problem, namely, how to induce faster transitions in multilevel quantum systems. In such context, a seemingly conflicting result is that such Rabi-like behavior produced by the moving walls involve more states as the oscillation amplitude grow, which compromises the fidelity when we want to drive the system to a specific level^6^. However, the well-known Rabi model suggests that larger amplitudes may induce faster oscillations between states which is certainly of interest for any application. Hence, a strategy that may speed up the dynamics overcoming such difficulty is highly desirable.

In the present report, we indeed overcome such difficulty and show how to drive the system from one quantum state to another in a relevant fraction of the quantum speed limit. To do so, we propose the use of two (or more) frequencies in the movements of the walls, in such a way that one frequency drives the system to the desired state (so it must be set at the resonance frequency) while the other one brings back the population from the higher levels. Hence, the resonance frequency must be calculated, with the corresponding Bloch-Siegert (BS) shift.

As an illustration of this procedure, we offer the transition from the ground state to the first excited state in a infinite square-well potential. An analysis of the robustness of the procedure is presented and contrasted with the small amplitude limit with the same fidelity. We conclude that our strategy is not only faster, but also increases the robustness of the method by strongly reducing the need for tuning the parameters to obtained a complete mode transfer, as discussed below. It is worth mentioning that our results resemble the unexpected behavior reported recently by Chang *et al*., as is explained below^9^.

This paper is organized as follows: after this Introduction, in Sec. 1 we discuss how to calculate the Bloch-Siegert shift in a multi-level quantum system, and present the results in the case of a square-well potential. In Sec. 2 we discuss how to achieve maximum population transfer using high amplitude movements in a multi-level system and use our approach to drive a system from the ground to the first excited state under an square-well potential. In Sec. 3 we test the robustness of the previous result. Finally, Sec. 4 closes the paper with our conclusions.

## Calculation of the Bloch-Siegert shift

The time evolution of the wave function of a particle of mass *m* under a one-dimensional harmonically moving potential $V(x,t)=V(x+\frac{d}{\omega }\,\cos (\omega t))$ is given by the time-dependent Schrödinger equation, namely

1 $$i\hslash \frac{\partial \psi }{\partial t}=[-\frac{{\hslash }^{2}}{2m}\frac{{\partial }^{2}}{\partial {x}^{2}}+V(x,t)]\psi .$$

In the comoving reference frame $q=x+\frac{d}{\omega }\,\cos (\omega t)$ the Eq. (1) reads

2 $$i\hslash \frac{\partial \psi }{\partial t}=[-\frac{{\hslash }^{2}}{2m}\frac{{\partial }^{2}}{\partial {q}^{2}}+V(q)+i\hslash d\,\sin (\omega t)\frac{\partial }{\partial q}]\psi ,$$

where $\frac{d}{\omega }$ is the amplitude of the oscillation, *ω* the frequency and $\hslash$ the reduced planck constant. Here we observe the equivalence between this kind of movement of the whole potential with an AC field at the quantum level, which is true also in the context of waveguides^10–12^. Consequently, all these settings are situations in which these ideas about controlling and speeding up the transition of states can be applied.

Expanding *ψ* in terms of the eigenstates *φ*_*n*_ of the unperturbed problem (*d* = 0),

3 $$\psi =\mathop{\sum }\limits_{n=1}^{\infty }\,{c}_{n}(t)\,{e}^{-i{E}_{n}t/\hslash }\,{\phi }_{n}(x),$$

where *c*_*n*_ is the probability amplitude and *E*_*n*_ the eigenenergy of the eigenstate *φ*_*n*_. Substituting the Eq. (3) into the Eq. (2), and projecting, we obtain

4 $$i\hslash \frac{d{c}_{n}}{dt}=-d\,\sin (\omega t)\mathop{\sum }\limits_{m=1}^{\infty }\,{e}^{-i{\omega }^{n,m}t}\,{p}_{n,m}\,{c}_{m}(t),$$

where ${p}_{n,m}=\langle {\phi }_{n}|\check{p}|{\phi }_{m}\rangle$ and *ω*^*n*,*m*^ = (*E*_*n*_ − *E*_*m*_)/$\hslash$, which is also the value of the resonance frequency between the state *n* and *m* in the limit *d* → 0. Here $\check{p}$ is the momentum operator. This equation can be solved numerically using an integrator.

As it was established by Shirley *et al*.^13^, the resonance frequency between levels *i* and *j* is achieved when the value of the time-averaged transition probability 〈*P*_*i*,*j*_〉, given by

5 $$\langle {P}_{i,j}\rangle =\frac{1}{T}{\int }_{0}^{T}\,|{c}_{j}(t){|}^{2}dt,$$

is maximum at fixed *d*. The value of 〈*P*_*i*,*j*_〉 can be calculated following the time evolution of the system starting from the state |*φ*_*i*_〉 through Eq. (4) for a sufficiently long time *T* to ensure convergence. Solving for the frequency *ω*_res_^*ij*^ that maximizes 〈*P*_*i*,*j*_〉, at a fixed value of *d*, we can calculate the BS shift for a resonance between the state *i* and *j* using Δ*ω*_BS_^*ij*^ = *ω*_res_^*ij*^ − *ω*^*ij*^.

In the case of an infinite square-well potential, namely

6 $$V(q)=(\begin{array}{ll}0, & {\rm{if}}\,0 < {\rm{q}} < {\rm{L}}\\ \infty . & {\rm{elsewhere}}\end{array},$$

follows that ${\phi }_{n}=\sqrt{2/L}\,\sin (n\pi x/L)$. At this point, it results convenient to introduce the adimensional variables: $\bar{t}=\frac{{\pi }^{2}\hslash }{2m{L}^{2}}\,t$, $\bar{\omega }=\frac{2m{L}^{2}}{{\pi }^{2}\hslash }\,\omega$, $\bar{d}=\frac{4mL}{{\pi }^{2}\hslash }\,d$, and ${\bar{p}}_{n,m}=\frac{L}{\hslash }{p}_{n,m}$. By doing so, the time evolution of the probability amplitudes is now given by

7 $$i\frac{d{c}_{n}}{d\bar{t}}=-\,\frac{1}{2}\bar{d}\,\sin (\bar{\omega }\,\bar{t})\mathop{\sum }\limits_{m=1}^{\infty }\,{e}^{-i({n}^{2}-{m}^{2})\bar{t}}\,{\bar{p}}_{n,m}\,{c}_{m}.$$

From now on, all the bar quantities are adimensional.

**Figure 1:**
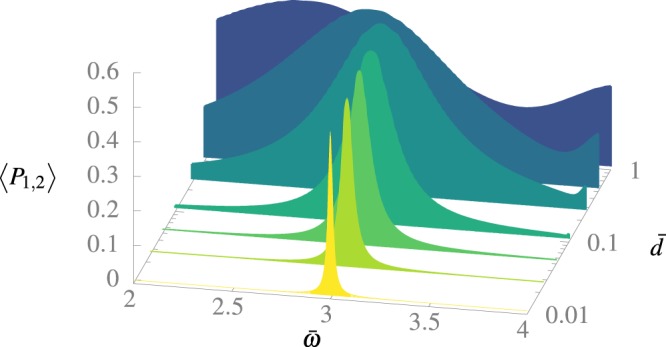
Time-averaged transition probability to the first excited state starting from the ground state for different values of *d̄* and *ω̄*. The numerical calculation was made by truncating the sum in Eq. (4) to *N*_*T*_ = 10 terms for an adimensional time of *T̄* = 1000, for every value of *d̄* and *ω̄*.

In Fig. 1 we observe the time-averaged transition probability from a starting state |*φ*_1_〉 to an objective state |*φ*_2_〉 as a function of *d̄* and *ω̄*. Note that a clear maximum is observed, and it becomes wider in frequencies as *d̄* grows. The BS shift is observed also in the displacement to lower frequencies of the maximum as we increase *d̄*. This result may seem at first sight curious because it is known that for a two-level system (TLS), the displacement goes to higher frequencies^14^, however in our case we see other levels that begin to participate as we increase *d̄*. This is an interesting and unexpected result, but beyond the scope of the present manuscript, that will be analyzed in a future work.

**Figure 2:**
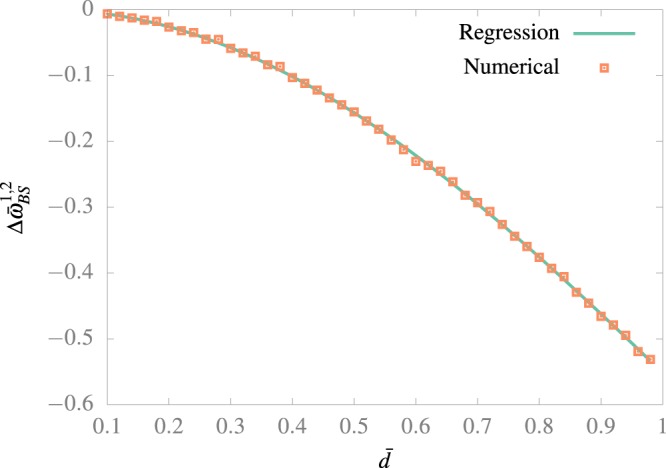
BS shift for a resonance between the ground and first excited state. The numerical calculation was made following 10 states for an adimensional time of *T̄* = 1000. The regression was performed using the model Δ*ω̄*_BS_^1,2^ = *ād̄*^2^ + *b̄d̄*^4^. Values of *ā* = −0.651231 and *b̄* = 0.09955 were obtained.

In Fig. 2 we show the results of a numerical calculation of the BS shift, following the methodology described above. As it was pointed out before, the resonance is now at lower frequencies. In order to obtain an expression of the BS shift for this particular system a polynomial regression was perform using the model Δ*ω̄* = *ād̄*^2^ + *b̄d̄*^4^, which gives the correct result at *d̄* = 0 and have the right parity. As it is seen in Fig. 2, the model agrees quite well with the results for the values of *ā* = −0.651231 and *b̄* = 0.09955.

## Maximum Population Transfer

Now we turn to our primary objective: achieve fast maximum population transfer. To do so, we make use of a system movement which is the superposition of two periodic movements, one to drive the system to the desired state and another to bring back the population from higher levels. Consequently, one movement must be set at the resonance frequency calculated in the previous section. However, it is not obvious what amplitude and frequency must have the second movement. Hence, as an illustration we will determine both parameters for the transition from the ground state to the first excited state in the case of a square-well potential. The dynamics are now given by

8 $$i\frac{d{c}_{n}}{d\bar{t}}=\frac{1}{2}[{\bar{d}}_{1}\,\sin ({\bar{\omega }}_{1}\,\bar{t})+{\bar{d}}_{2}\,\sin ({\bar{\omega }}_{2}\,\bar{t})]\times \mathop{\sum }\limits_{m=1}^{{\rm{\infty }}}\,{e}^{-i({n}^{2}-{m}^{2})\bar{t}}{\bar{p}}_{n,m}\,{c}_{m}.$$

As we established before, we must set *ω̄*_1_ = *ω̄*_res_^1,2^(*d̄*_1_) and the values of *d̄*_2_ and *ω̄*_2_ remain to be solved, for a fixed value of *d̄*_1_.

A first approach to the solution could be derived from the following argument: the first frequency drive the system from the ground state to the first excited one, but carrying also some population to higher states, mainly the second excited state. As a first approximation the solution for *ω̄*_2_ and *d̄*_2_ should satisfy *ω̄*_2_ = *ω̄*_res_^2,3^(*d̄*_2_), because at this frequency the population is transferred between the first excited state and the second one, and so the value of *d̄*_2_ remains to be found. However, we expect that only a small (but relevant) fraction of the population is in the second excited state during the process, so that it is not a strict requirement to set *ω̄*_2_ just at the resonance. For the same reason, we expect a small value of *d̄*_2_. In other words, it is enough that *ω̄*_2_ is near *ω̄*_res_^2,3^(*d̄*_2_) ≈ *ω*^2,3^, which gives us a quite useful idea where to start to tune the system up.

**Figure 3:**
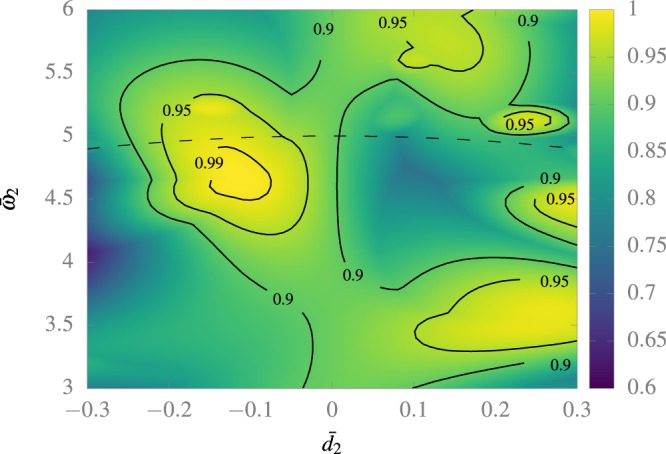
Maximum population transfer to the first excited state starting from the ground state for different values of *d̄*_2_ and *ω̄*_2_, and *ω̄*_res_^2,3^(*d̄*_2_) as the dashed line. The numerical calculation was made following 10 states for a time of *T̄* = 1000.

As an example of our approach, let us consider a value of *d̄*_1_ = 0.754. It follows from the calculations of the previous section that the resonance between the ground and first excited state is at *ω̄*_1_ = *ω̄*_res_^1,2^(*d̄*_1_). Now, we must tune *ω̄*_2_ and *d̄*_2_. In the Fig. 3 we show the maximum population transfer for different values of *d̄*_2_ and *ω̄*_2_, near the resonance frequency. We can observe a clear and wide maximum located at *d̄*_2_ = −0.114 and *ω̄*_2_ = 4.630. As expected, the optimal value of *ω̄*_2_ is bellow *ω̄*^2,3^.

**Figure 4:**
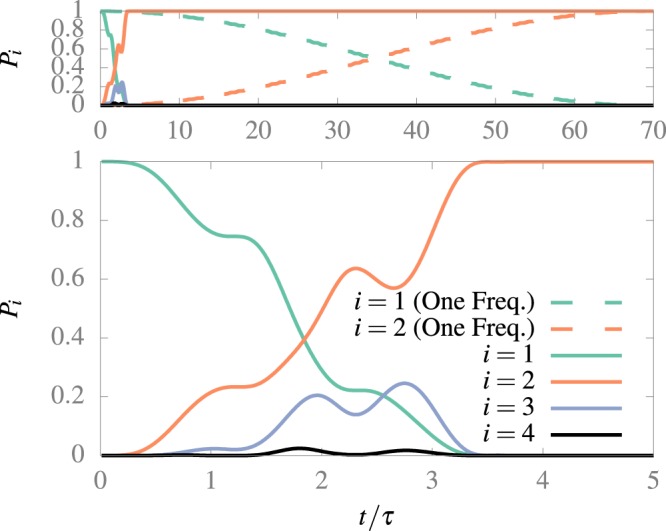
Comparison between the transitions between the ground and first excited state obtained with single frequency movement (dashed line) and two frequency movement (continuous line). At the top, we show the dynamics at large times. The numerical calculation was performed following 10 states. Both transitions reach 99.97% of fidelity. The driver used in the dashed one are set at *d̄* = 0.034 and *ω̄* = 2.99925. The drivers used in the continuous one is set at *d̄*_1_ = 0.754, *d̄*_2_ = 0.114, *ω̄*_1_ = 2.662, and *ω̄*_2_ = 4.630.

In Fig. 4 we observe the transition produced with the optimal values encountered while, at the top of the figure, a comparison with the slower transition that reaches the same population transfer to the first excited state but only with one driving movement of amplitude *d̄* = 0.034 and frequency *ω̄* = *ω̄*_res_^1,2^(*d̄*). Both transitions have a fidelity of 99.97%, and so they are comparable. Note that we can observe a ladder-type transition structure in the probabilities, as is expected at high amplitude^15^. Note that for the two frequencies the transition is about 20 times faster.

Now we can compare our transition times with the quantum speed limit^16^. The minimum time where a quantum transition can be performed is given by

9 $${t}_{{\rm{\min }}}=\frac{\hslash \pi }{\Delta E},$$

where Δ*E* is the energy gap between the initial and final state. The transition presented in Fig. 4 is performed in *t*_our_ = 3.5*t*_min_, thus approaching the quantum speed limit. Recent approaches to the two-level version of the problem, such as FIESTA^17^, where the amplitude of an AC field at the resonance frequency is modulated, achieve transitions in *t*_FIESTA_ = 2.4*t*_min_ with a fidelity of 99.98%. Of course in our case we appeal to the existence of more energy levels to speed up the transition, so our result is not only conceptually different from the TLS approach, but it is also more robust as we will now analyze.

An alternative approach for solving this problem, at least approximately, is to assume that *ω̄*_2_ = *nω̄*_1_, with *n* an integer, and then use a Floquet approach to solve for *n*, *d̄*_1_, *d̄*_2_, and *ω̄*_1_. Indeed, according to Fig. 3 such an approach is expected to lead to results with at least 95% of fidelity, assuming the same value of *ω̄*_1_ that we obtained. Hence, such an alternative approach should provide a result that is quite close to the solution that we found.

## Robustness

**Figure 5:**
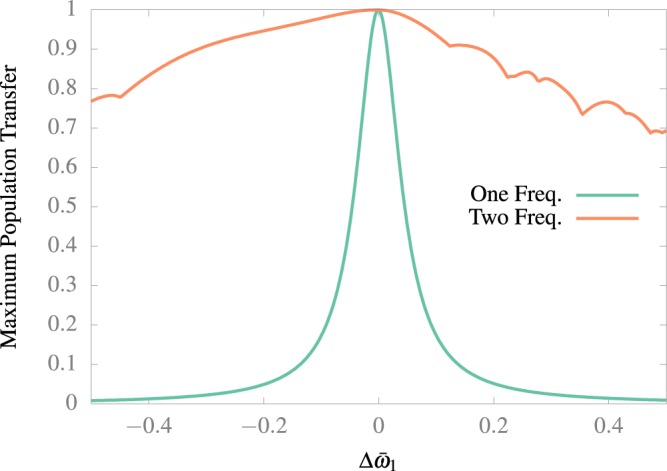
Comparison of the maximum population transfer from the ground to the first excited state as a function of detuning, obtained with one and two frequency movements. The numerical calculation was performed following 10 states.

**Figure 6:**
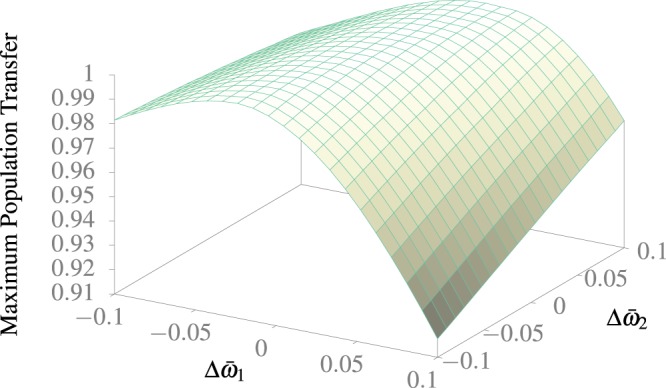
Maximum population transfer from the ground to first excited state as a function of detuning of both frequencies. The numerical calculation was performed following 10 states.

One of the main possible issues of setting up a real application is the need to tune up the precise frequency to observe such an effect. Hence it is desirable to have a robust behavior under frequency detuning. In order to test that, in Fig. 5 we compare the maximum population transfer under different detuned frequency for the optimal values encountered in the previous section and its counterpart using just one driving frequency. We can observe that the transition is far more robust with two frequencies instead of one. In Fig. 6 we compare the effect of a detuning in *ω̄*_1_ and *ω̄*_2_ from the optimal values. As we can see the process can become more robust with the introduction of a second driving frequency.

Counter-intuitively, the transition process using two frequencies is far more robust than with just one frequency. This comes from the fact that the use of two frequencies allows us to use larger amplitudes, which have a wider spectrum of resonance frequencies.

## Conclusions and Discussion

Summing up, we show how to speed up maximum population transfer in a multi-level quantum system. To do so, we make use of two frequency movements, with one frequency at a high amplitude and the second specifically designed to overcome the inherent difficulties when such amplitude is applied to multi-level quantum system. This approach allows us not only to speed up the transition to times in a quantum square-well potential close to the quantum speed limit, with a fidelity of 99.97% in comparison with a case that uses just one frequency; but also to make the transition more robust by strongly reducing the sensibility of the parameters, frequency and amplitude, required to tune-up the transition, which confirm the unexpected conclusion presented very recently by Chang *et al*.^9^ for a multilevel system.

In comparison with an approach designed for TLS systems, such as FIESTA^17^, the fidelities we obtained are equivalent while the transition times are similar but a little bit slower. However, we must recall that our work deals with the inherent nature of multi-level system instead of a TLS, so that we don’t require to establish amplitude restrictions, and therefore, it may have a wider applicability. Nevertheless, given that both approaches are not incompatible, it may be worth to seek a mixed approach. Finally, we must remark the simplicity of our approach, where only two parameters remain to be found instead of the four of FIESTA. This, along with the improvement in robustness discussed above, should lead to a feasible experimental implementation.

In the future, we would like to apply a similar strategy to increase the robustness of other effects like coherence destruction of tunneling, dynamic localization, reduction of scattering, etc. The approach could also be used to speed up other quantum transitions process, like molecular isomerization, etc. Along the same lines, it would be of interest to study the implementation of our protocol in many-body systems, especially how it relates with interesting many-body phenomena such as dynamical many-body freezing^18,19^, that has been observed recently in Ising chains at certain driving frequencies^20^. This is particularly interesting, as such phenomena could limit the frequencies that can be used to control the state of the system, and will be analyzed in a future manuscript. Another interesting phenomena is the so-called many-body localization, where a transition between localized to delocalized state can be produced by a single frequency driving^21,22^. This interesting result brings the questions as to whether a protocol like the one presented here can speed up such process? Finally, it has been recently demonstrated the emergence of chaotic behavior in interacting many-body systems^23,24^. Moreover, it has been shown very recently that it can be suppressed if the driver strength is above a certain threshold^25^, which again shows the relevance of developing protocols that work in the strong amplitude regime, like the one we consider in the present manuscript.

Our results could be tested experimentally in photonic lattices, where Rabi oscillations have been observed using moving potentials, namely, a lattice that is transversely modulated in the perpendicular direction with an additional periodic refractive index^26^. Hence, in our case, the modulation must be the sum of two additional refractive indexes, which is doable with the current experimental capabilities.
